# Cerebral Tuberculosis in a Patient Following Treatment With Infliximab for Ankylosing Spondylitis: A Case Report

**DOI:** 10.7759/cureus.39117

**Published:** 2023-05-17

**Authors:** Ioana Cretu, Corina Geoanta, Oana-Irina Bogheanu, Mihaela Milicescu, Mihai Bojinca, Mihai Costache, Catalin Cirstoiu, Bogdan Cretu

**Affiliations:** 1 Internal Medicine & Rheumatology, Doctor Ion Cantacuzino Clinical Hospital, Bucharest, ROU; 2 Orthopedics & Traumatology, Bucharest University Emergency Hospital, Bucharest, ROU

**Keywords:** biological therapy, treatment, hip, ankylosing spondylitis, tuberculosis

## Abstract

Ankylosing spondylitis (AS) mainly belongs to the group of axial spondylitis. It is a chronic inflammatory disease that primarily affects the spine, but can also affect peripheral joints. It is characterized by inflammatory lower back pain and morning stiffness. Tuberculosis is still a cause of morbidity and mortality in developing countries. Management of patients with AS consists of patient education, spinal mobility exercises, non-steroidal anti-inflammatory drugs (NSAIDs), corticotherapy, and anti-tumor necrosis factor alpha (TNF-α) biological agents. Anti-TNF-α biological agents have changed the prognosis of patients with AS. They contain anti-TNF-α monoclonal antibodies (golimumab, infliximab, adalimumab, certolizumab) and the soluble TNF-α receptor (etanercept). Hip and knee involvement is common in patients with AS, as evidenced in radiographs as bone erosion and joint space narrowing. The patient may have severe pain, stiffness, and loss of mobility, and the treatment involves surgery for joint arthroplasty. We present the case of a 63-year-old patient with axial spondyloarthritis who was treated with infliximab and developed cerebral tuberculosis after three years of biological therapy. The purpose of the study is to determine the possibility of resuming biological therapy at the time of AS reactivation, given the long-term treatment and adverse reactions of cortisone therapy (aseptic necrosis of the femoral head).

## Introduction

Ankylosing spondylitis (AS) is part of the predominantly axial group of spondylitis. It is a chronic inflammatory disease that mainly affects the spine, but can also affect the peripheral joints. Genetic factors play an important role in the onset of the disease; the human leukocyte antigen (HLA)-b27 pattern is found in 90% to 95% of patients while the incidence of HLA-B27 in the general population is about 8%. The prevalence is three times higher in men than in women [1]. Ankylosing spondylitis is characterized by the appearance of inflammatory lower back pain and morning stiffness. Over time, the mobility of the spine decreases. The evolution is variable, from mild pain to total ankylosis. Hip and knee involvement is common in patients with AS; the reported prevalence of clinical hip involvement in AS is from 24% to 36% and the prevalence of radiographic hip arthritis ranges from 9% to 22% [2-4]. Unlike new bone formation in the axial spine, synovial inflammation within the hip joint causes bone erosion and joint space narrowing which is treated with joint arthroplasty [5-10]. Also, tuberculosis (TB) remains a cause of morbidity and mortality in underdeveloped countries. Central nervous system (CNS) damage occurs in approximately 2% to 5% of TB patients in endemic regions. Manifesting as meningitis, cerebritis, and tuberculous abscesses or tuberculoma, it occurs in approximately 1% of TB patients. In endemic regions, tuberculomas account for as many as 50% of all intracranial masses. Clinical manifestations are usually accompanied by signs and symptoms of focal neurological deficits (altered sensorium, hemiparesis, fever, meningism) [11-15].

Patients with AS receive counseling, physiotherapy for spinal mobility, and nonsteroidal anti-inflammatory drugs (NSAIDs) as the first-line treatment; continuous treatment with NSAIDs is recommended as a first step for symptomatic, persistently active disease [1]. The use of corticotherapy should be used only for short periods given the risk of developing side effects (musculoskeletal: osteoporosis, myopathy, osteonecrosis; ophthalmologic: glaucoma, cataracts; cardiovascular: hypertension, dyslipidemia, edema; infectious: viral, skin infections; gastrointestinal: peptic ulcer; psychiatric: mood disorders; endocrine and metabolic: diabetes, obesity; dermatological: infections, atrophy, hirsutism, alopecia). Compared to other drugs used in rheumatology, the use of glucocorticoids is associated with a low incidence of short-term side effects, which make them all the more common and more severe when the corticosteroid therapy is used for longer and with higher doses [5].

Anti-tumor necrosis factor alpha (TNF-α) biological agents have changed the prognosis of patients with AS. These agents comprise anti-TNF-α monoclonal antibodies (infliximab, adalimumab, golimumab, certolizumab) and the soluble TNF-α receptor (etanercept). Their early use in forms of non-radiographic axial spondylitis, prior to bone erosion, can prevent the appearance of syndesmophytes and other osteoproliferations [1]. Toxicities associated with anti-TNF-α administration include skin damage at the injection site, increased risk of infection (tuberculosis, fungal infections), increased risk of developing neoplasms (lymphomas), risk of worsening heart failure (treatment is contraindicated in patients with stage III or IV heart failure), risk of demyelinating disease, and risk of immunogenicity, with the appearance of antibodies directed against the biological agent. Patients should be tested for hepatitis B virus (HBV), HCV, and tuberculosis before initiating biological treatment [1].

A new anti-interleukin (IL)-17A biological agent, secukinumab, was recently approved. The IL-17A, a member of the IL-17 family, is a cytokine involved in inflammatory and immunological responses and has been shown to play an important role in the pathogenesis of AS. Secukinumab is an option for treating active AS in adults whose disease responded inappropriately to NSAIDs or anti-TNF-α [11].

This article describes the onset of TB with cerebral determinations in a patient with AS, after the initiation of biological therapy (infliximab). The purpose of the study is to determine the possibility of resuming biological therapy at the time of AS reactivation, given the long-term treatment and adverse reactions of cortisone therapy (aseptic necrosis of the femoral head). In order to manage the case, a close collaboration between different medical specialties was necessary (rheumatology, pneumology, infectious diseases, orthopedics).

## Case presentation

A 63-year-old male patient, known to have axial spondyloarthritis for about 30 years was treated with infliximab from June 2010 to November 2013. He developed cerebral TB after three years of biological therapy.

The patient has no family history of TB and spondylitis. In March 2010 the patient presented bilateral sacroiliitis grade II III, positive HLA-B27, and inflammatory syndrome. Axial spondyloarthropathy was diagnosed in 1990 and the treatment with sulfasalazine began in January 2010. Given the axial form of the disease and the persistence of clinical and biological activity of the disease under treatment, the quantiferon gold TB test was performed on the recommendation of the multidisciplinary team. The test was positive and the patient began prophylactic treatment with isoniazid for nine months. Between March and May 2010, the patient had a steady evolution. 

In May 2010, he was administered the first infusion of infliximab. In October 2010 he was hospitalized for an infliximab infusion, but he was diagnosed with acute cholecystitis (pain in the right hypochondrium, nausea, vomiting, biological inflammatory syndrome) with symptomatic remission. He was recommended a cholecystectomy in a month. The cholecystectomy was made in January 2011.

**Figure 1:**
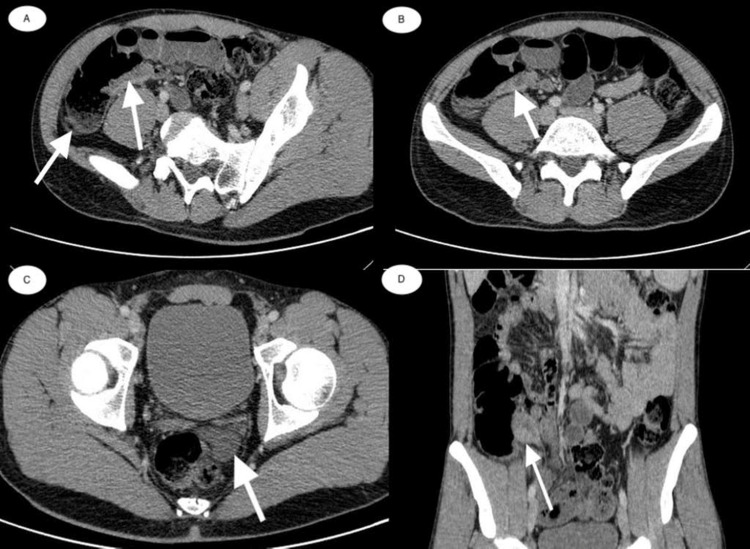
Contrast CT scans of the abdomen and pelvis A paracecal inflammatory area is noted in the right iliac fossa (A, B, and D) that is found all along the loop of the terminal ileum, measuring about 10 x 15 cm. It is determined by the highlighting of its wall as well as of the cecal wall. It is accompanied by intraperitoneal fluid in a small quantity that occupies the bottom of the Douglas sac and the perivesical sacs (C).

In February 2012, the patient complained of lower abdominal pain (colicky pain), which started five days before hospitalization and it progressively accentuated. Contrast CT of the abdomen and pelvis was performed (Figure 1). He was hospitalized for colonoscopy and biopsy, with intestinal TB being considered or Crohn's disease.

In 2012, the colonoscopy examination of the terminal ileum and the biopsy were within normal limits. Two small/medium biopsy fragments from the terminal ileum were sent to histopathological examination and the following was observed: preserved villous architecture, mucosal epithelium intact with no architectural changes, no villous atrophy, no pathological inflammatory infiltrates, and no other microscopic changes. Considering the normal aspect of the abdominal CT examination and normal values of the tests, the episode was classified as acute appendicitis with appendicular plastron, remitted under antibiotic treatment.

**Figure 2:**
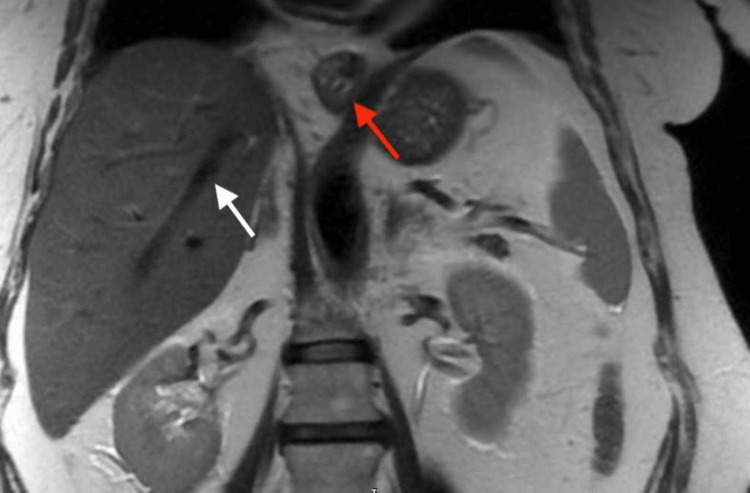
MRI with a contrast agent Transhiatal gastric hernia (red arrow) without intrahepatic and extrahepatic bile duct dilations and with a lumen of normal thickness (white arrow).

In November 2013, the patient presented with inflammatory syndrome without clinical correlation (preserved joint mobility, absence of painkillers), hepatic cytolysis syndrome, and cholestasis syndrome. A cholangiography-MRI with a contrast agent was performed (Figure 2).

**Figure 3:**
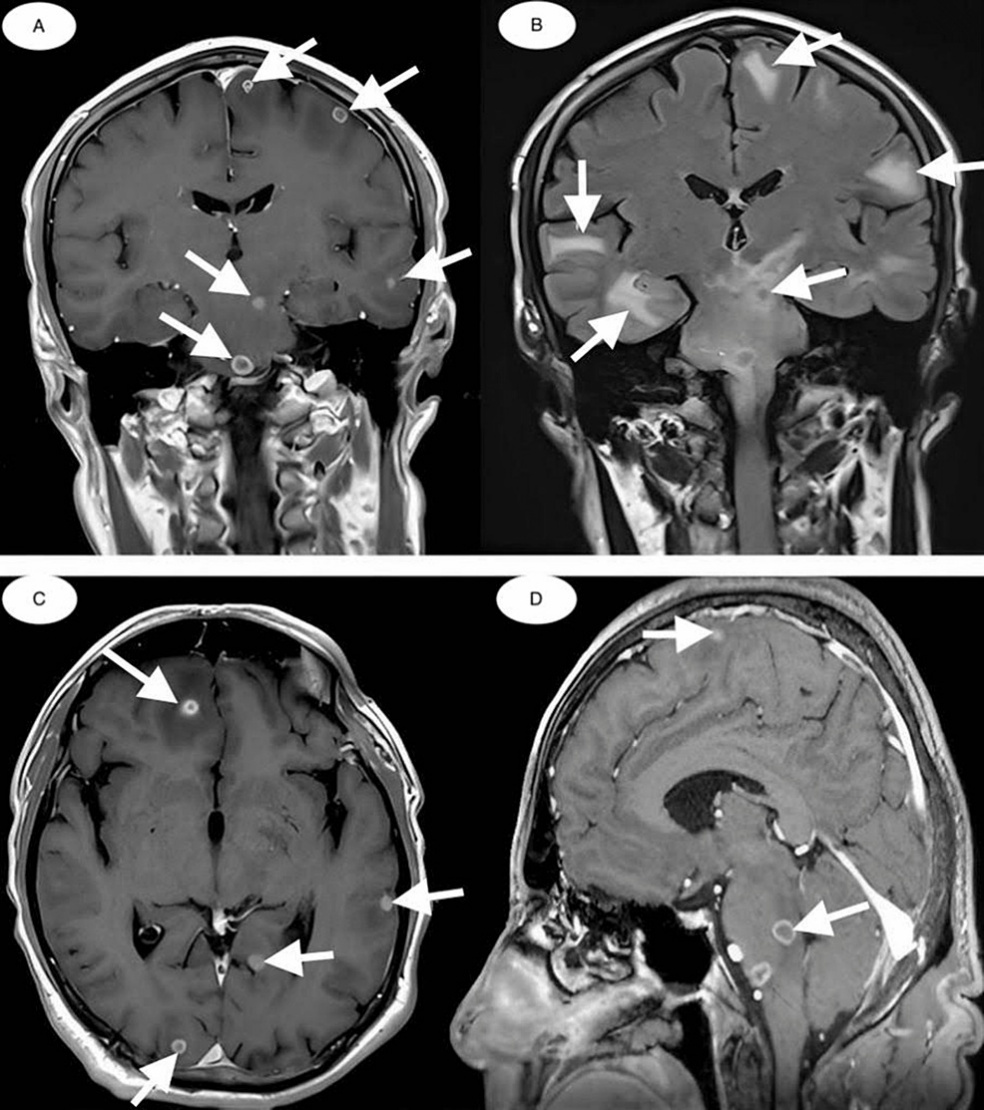
Brain CT scans in different planes (coronal, axial, and sagittal) Multiple iodophile neoplastic lesions (A, C, and D) are identified with centimetric diameters. They are located diffusely throughout the brain mass and accompanied by perilesional edema (B) in the cerebral trunk (secondary determinations).

In December 2013, he presented with an altered general condition, fatigue, fever, profuse sweating, dizziness, diplopia in both eyes, and pain in the right iliac fossa and right hip region. Native chest CT and pelvic CT (lower abdomen) with contrast showed aspects within normal limits. Native brain CT and contrast agent revealed multiple iodophile neoplastic lesions and perilesional edema in the cerebral trunk (Figure 3). The patient was transferred to the infectious diseases hospital for a spinal puncture and cerebrospinal fluid (CSF) cultures.

In September 2014, the diagnosis of TB was confirmed by cerebral determinations followed by histopathology. The histopathological examination suggested TB infection by the presence of a typical granulomatous reaction consisting of uninucleated epithelioid cells and giant cells (Langhans type) mixed with small lymphocytes around a central necrotic area (caseating necrosis). Adjacent brain tissue showed reactive changes: mild perivascular lymphocytic and histiocytic infiltration, microgliosis, and reactive astroglia (Rich foci).

This presented clinical and biological reactivation (inflammatory back pain, C-reactive protein (CRP)=40.72 mg/L) in the context of giving up corticosteroid therapy (initiated after cessation of infliximab). Treatment was reinitiated medrol 4mg/day plus sulfasalazine in a progressive dose of up to 2 g/day.

Over the years, as biological therapy with anti-TNF-α (pneumology decision) could not be restarted, the patient was administered corticosteroid therapy to control AS. In September 2020, the decision was made to introduce a low dose of methotrexate (2.5 mg 2 cp/week), in addition to treatment with sulfasalazine 6 cp/day.

In 2021, the patient presented with severe pain in the hip joints. Symptomatology began progressively. He was diagnosed with bilateral femoral head osteonecrosis, determined by the long treatment with corticosteroids but also by the inefficient control of AS. Given the disabling symptoms, the patient was registered in the orthopedic clinic and the decision was made for bilateral hip arthroplasty. In March 2021, surgery was performed and the uncemented prosthesis was placed via a minimally invasive approach at the level of the right hip. Subsequently, five months later, surgery was performed on the left hip.

The symptoms of the 2013 episode (altered general condition, fever, fatigue, diplopia, dizziness) are nonspecific, and the differential diagnosis could be made with cerebral toxocariasis, brain metastases (also suggested by brain CT with contrast agent), especially considering that the main adverse reactions of biologic therapy with anti-TNF-α are the increased risk of infections and neoplasms. Confirmation of the diagnosis of cerebral TB was made by histopathology and tuberculostatic treatment was initiated with a favorable evolution.

To summarise, in this case, anti-TNF-α therapy (infliximab) was initiated and then stopped when cerebral TB appeared at which point tuberculostatic treatment was begun. After that, to control the disease activity, the patient was administered sulfasalazine in a progressively increased dose along with corticosteroid therapy. Following long-term glucocorticoid therapy, the patient was diagnosed with aseptic osteonecrosis of the bilateral femoral head, for which bilateral hip arthroplasty was performed. Subsequently, low-dose methotrexate was added. At this stage, AS was well-controlled and showed no signs of reactivation.

## Discussion

The first-line of medication for spondyloarthropathies include NSAIDs, corticosteroids (to address swelling in one specific area of the joint), disease-modifying anti-rheumatic drugs (methotrexate and sulfasalazine) for peripheral arthritis, and TNF-α blockers known as biologics that can treat both spine and joint arthritis.

Tuberculosis of the central nervous system can complicate the course of patients treated with anti-TNF-α agents, which is why regular evaluation is essential. In patients with an increased risk of developing or reactivating TB, non-anti-TNF-α agents may be a safer method of treatment. The mechanism of action and safety profile from clinical trials indicate that secukinumab is a safe option in those at risk of TB reactivation and may be a good therapeutic option for patients with spondyloarthropathies who are at high risk of developing TB.

**Figure 4:**
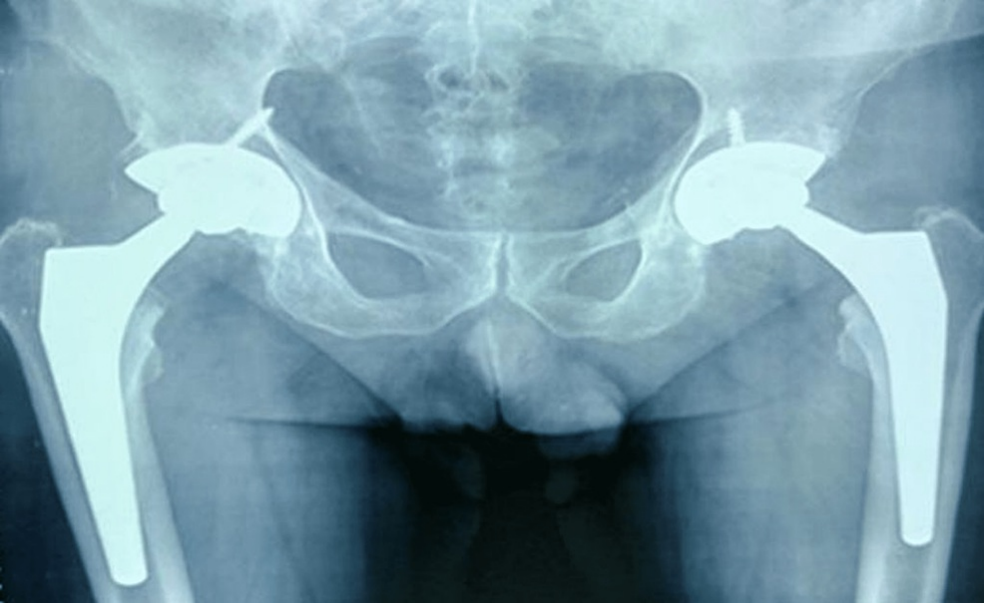
Anterior-posterior view radiograph shows our patient's bilateral hip arthroplasty

The problem raised by our case was how to safely resume the treatment for the patient's AS without clinical reactivation of TB. We administered corticotherapy but the patient had side effects such as aseptic osteonecrosis of the femoral head, which required bilateral hip replacement (Figure 4).

When anti-TNF-α is administered, the risk of TB reactivation is high, so non-TNF-α biological agents such as anti-IL-17A antibodies (secukinumab), may be considered [16-20]. According to a recent study, secukinumab is not associated with an increased risk of TB reactivation. This study was performed to examine the association of secukinumab treatment with TB reactivation in 12,319 patients with moderate to severe psoriasis, active psoriatic arthritis, or AS; out of whom TB was present in only 13 patients (seven of the 13 had new cases of TB) [5].

## Conclusions

The risk of TB in patients with biological treatment is high. Screening for infections (hepatitis B, hepatitis C, TB) is required before initiating biological treatment. And for those who test positive for these infections, biological treatment with anti-IL-17A (secukinumab) may be administered as it does not present the risk of reactivation of TB, and its use is encouraged for the treatment of AS. Secukinumab is effective and well tolerated for the treatment of adults and it is a useful treatment option for patients who have an inadequate response to or are intolerant of TNF inhibitors. 

In conclusion, we believe secukinumab is the best option for a patient who runs the risk of reactivating AS. All the decisions regarding the management of complications, treatment of adverse reactions, and overall management should be taken within a multidisciplinary team to improve patient outcomes and avoid possible aggravation.
